# Breed-specific enteric methane emission factor assessment in Italian dairy cattle leveraging DHI and primary ration data

**DOI:** 10.1093/jas/skag171

**Published:** 2026-06-02

**Authors:** Giulia Ferronato, Mesfin Mekonnen Moliso, Raphael Mrode, Paolo Ajmone-Marsan, Riccardo Negrini

**Affiliations:** Department of Civil, Environmental, Architectural, Engineering and Mathematics (DICATAM), Università degli Studi di Brescia, 25121, Brescia, Italy; Department of Animal Science, Food and Nutrition (DIANA), Università Cattolica del Sacro Cuore, 29122, Piacenza, Italy; Scotland’s Rural College, Easter Bush, EH25 9RG, Edinburgh, United Kingdom; Department of Animal Science, Food and Nutrition (DIANA), Università Cattolica del Sacro Cuore, 29122, Piacenza, Italy; Department of Animal Science, Food and Nutrition (DIANA), Università Cattolica del Sacro Cuore, 29122, Piacenza, Italy

**Keywords:** enteric methane, dairy cow, feeding strategy, IPCC

## Abstract

Enteric methane (CH_4_) emissions from dairy cattle are a major contributor to greenhouse gas footprint in the livestock sector. This study applied Intergovernmental Panel on Climate Change (IPCC) Tier 2 equations to estimate enteric methane emissions in Italian Holstein, Brown Swiss, and Red Pied herds using either default dietary assumptions or farm-specific diet information. By combining individual Dairy Herd Improvement (DHI) test-day records with primary ration data, the analysis examined how improved input data resolution affected methane estimates across breeds and animal categories under commercial farming conditions. Data were collected from 138 Italian dairy farms participating in the national Dairy Herd Improvement (DHI) program between January 2021 and December 2022, with farm rations recorded concurrently during DHI test days by trained technicians using a standardized questionnaire. Breed, emission estimation approach, and their interaction were analyzed using aligned rank transform (ART) procedures with permutation-based *P*-values. Holstein cows exhibited greater daily methane production (MeP = 443.60 ± 5.68 g/d; *P* < 0.001), whereas Red Pied herds showed the greatest methane intensity (MeI = 29.99 ± 0.70 g/kg fat- and protein-corrected milk; *P* < 0.001). Breed and CH_4_ estimation approaches affected emission estimates, however, their interaction was not significant, indicating consistent breed rankings across methods. Post hoc analyses revealed no significant differences in methane indices among breeds. Incorporation of farm-specific ration data impacted emission estimates, particularly for nonproductive groups such as dry cows and heifers, highlighting the importance of context-specific dietary inputs for improving the accuracy and representativeness of CH_4_ inventories.

## Graphical abstract

**Graphical Abstract:**
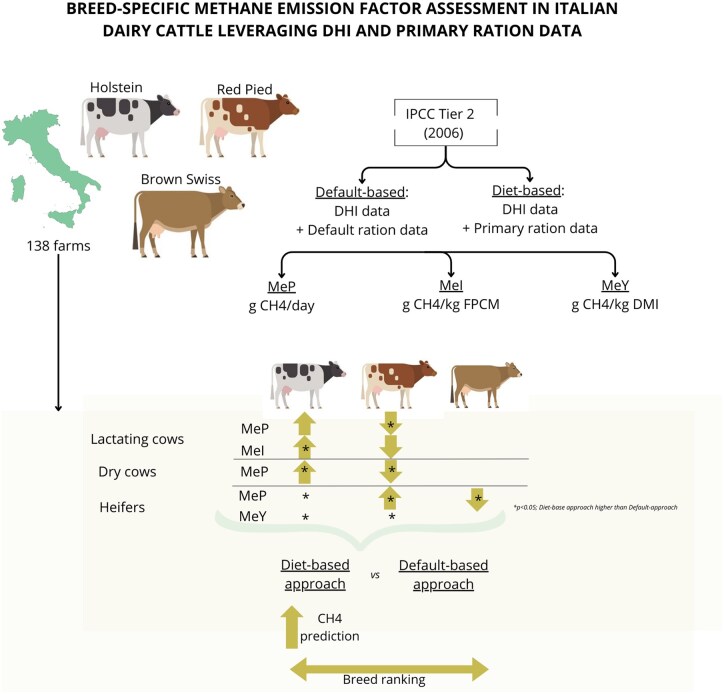

## Teaser

- Holstein, Brown Swiss, and Italian Red Pied cows differed markedly in milk yield, methane production and methane intensity revealing productivity-efficiency trade-offs among breeds.
- Replacing default-based diet parameters with diet-specific values derived from farm-level ration information on markedly shifted emission estimates, especially for dry cows and heifers, highlighting the importance of accurate diet inputs in robust CH_4_ modelling.

## Introduction

Enteric methane (CH_4_) emissions from dairy cattle represent a major challenge for the environmental sustainability of livestock systems, given their high global warming potential relative to carbon dioxide over a short time horizon (Gerber et al. 2013; Correddu et al. 2023; del Prado A et al. 2023). In dairy production, enteric fermentation is the dominant source of methane emissions, whereas manure management contributes a smaller proportion (Dämmgen et al. 2013). The magnitude of enteric CH_4_ emissions is influenced by multiple interacting factors, including animal genetics, physiological stage, and diet composition, with feed intake and energy supply playing a central role (Haque 2018; Bougouin et al. 2022). Breed-related differences add further complexity to methane assessment. High-yielding dairy breeds such as Holstein typically exhibit greater methane production per animal, largely as a consequence of higher dry matter and energy intake, whereas dual-purpose breeds generally show lower absolute emissions but often higher emissions per unit of product (Beauchemin et al. 2020; Rivero et al. 2021). Distinguishing these breed-specific patterns is essential for interpreting emission estimates and for supporting mitigation strategies that balance productivity and environmental performance. At the scale of national inventories and territorial assessments, enteric methane emissions are commonly estimated using empirical models, including those proposed by the Intergovernmental Panel on Climate Change (IPCC). These models are widely applied because of their simplicity, transparency, and low data requirements, and they provide harmonized estimates that are comparable across countries (IPCC 2019). However, empirical approaches rely on standardized assumptions regarding feed intake and diet composition, which may not adequately capture on-farm variability across breeds, production systems, and animal categories (Alemu et al. 2011; Danielsson et al. 2017; Liu et al. 2022; Colombini et al. 2023). As a result, excessive standardization may obscure meaningful differences in CH_4_ emissions under heterogeneous commercial conditions.

Improving the accuracy of CH_4_ estimates requires integrating context-specific information while maintaining methodological consistency. Originally developed for genetic evaluation and performance monitoring, Dairy Herd Improvement (DHI) recording systems generate harmonized, high-resolution longitudinal data on milk production, composition, and herd structure, representing a valuable but underutilized resource for refining enteric methane estimates and other sustainability indicators at the animal, herd, and sector levels (Christen A-M et al. 2022; ICAR 2024; ICAR 2006; Sejian et al. 2011). When aligned with IPCC Tier 2 methodologies and supplemented with farm-level dietary information, DHI data have the potential to improve the realism and precision of emission estimates at both the individual-animal and herd levels, while remaining scalable for inventory purposes.

Accordingly, the objective of this study was to evaluate breed-specific CH_4_ emissions in Italian Holstein, Brown Swiss, and Red Pied dairy herds by comparing methane estimates through IPCC equations and based on default dietary assumptions or actual diet data. By integrating individual DHI test-day records with primary dietary information, this study assesses how the resolution of input data affects methane emission magnitude, variability, and breed ranking across animal categories, providing insight into the added value of diet-based approaches for methane quantification under commercial conditions.

## Materials and methods

### Data collection

The study focused on the three main dairy breeds in Italy: Frisona Italiana (Holstein), Bruna Italiana (Brown Swiss), and the double-purpose Pezzata Rossa Italiana (Italian Red Pied). DHI data were obtained from 138 Italian commercial farms: 97 Holstein (6664 lactating cows), 6 Brown Swiss (380 lactating cows), and 35 Red Pied (1128 lactating cows) monitored by the Italian Breeders Association (AIA) between January 2021 and December 2022. The DHI dataset included test-day records of milk yield, fat and protein content, and herd demographic structure. Data were collected nationwide in accordance with International Committee for Animal Recording (ICAR) protocols by certified milk-recording technicians, specifically trained and routinely quality-controlled to ensure standardized and reliable field measurements. Feed management data were collected through a standardized survey administered by trained technicians during a single farm visit. The survey captured detailed information on daily ration composition and the amount of each feed ingredient supplied, disaggregated by animal category (lactating cows, heifers, and dry cows). Ration data were analyzed using Razio-Best software (version 5.60) to evaluate nutrient composition (Calamari 2002). Daily milk yield (kg) was converted to daily Fat- and Protein-Corrected Milk (FPCM) using the equation recommended by the International Dairy Federation (IDF 2015), as shown below:


(1) FPCM(kg/day)=milk yield(kg/day)×(0.1226×fat%+0.0776×protein%+0.2534)

### Enteric methane estimation

Per capita daily enteric CH_4_ emissions were estimated using the Tier 2 methodology outlined by the Intergovernmental Panel on Climate Change (IPCC 2019), as shown in Equation 2:


(2) $${\text{CH}}_{4}\left(g/\text{head}/\text{day}\right)=\left(\text{GE}\times \text{Ym}\right)/55.65$$

where GE is the Gross Energy (MJ/d) and Ym the CH_4_ conversion factor.

**Figure 1:**
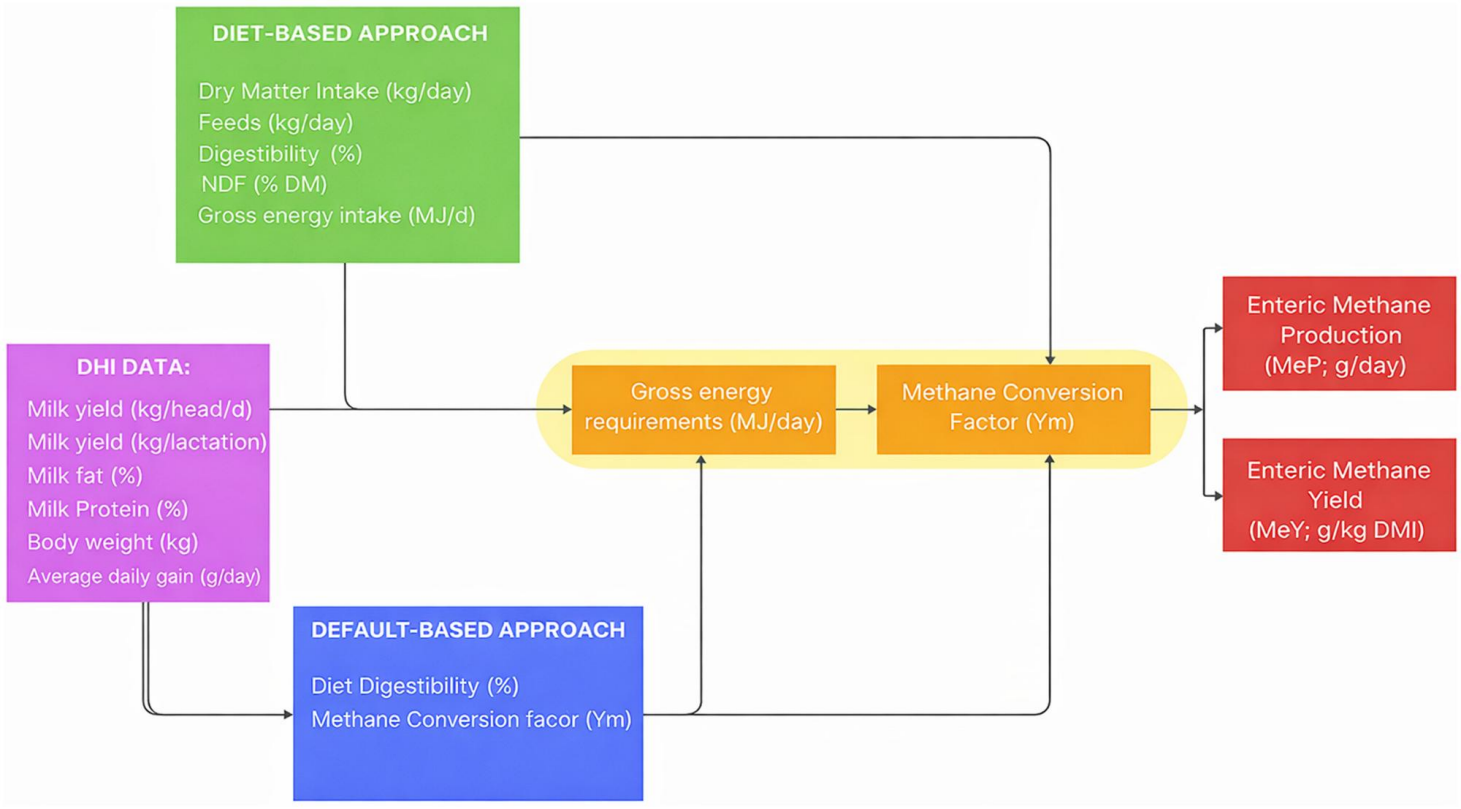
Overview of the methodological framework adopted for the estimation of enteric methane emissions. The approach includes two methods: a default-based approach using IPCC reference values diet parameters and a diet-based approach grounded in actual ration composition data.

Two distinct modelling approaches were employed to apply this equation (Figure 1):

1. Default-based approach, which relied on dietary parameters from established national and international guidelines, and
2. Diet-based approach, which utilized primary ration composition data collected for each animal category.

In both approaches, key animal parameters, body weight (BW), mature weight (MW), and average daily weight gain (ADG), were assigned based on breed and physiological class (Table 1) by authors’ personal estimation from data obtained during extension service activity. For lactating cows, daily milk yield, fat content, and protein content were retrieved from DHI test-day records.

**Table 1:** Standard reference values for body weight, mature weight, digestible energy (DE), neutral detergent fiber (NDF) and gross energy intake (GEI) for lactating cows, dry cows and heifers of the three main dairy breeds in Italy: Holstein, Italian Red Pied and Brown Swiss. Data are presented as mean ± standard error. Significant differences between breeds are indicated by superscript letters (*P* < 0.05).

Animal category	Parameters	Measure units	Breeds			*P*-value
			Holstein	Italian Red Pied	Brown Swiss	
**Lactating cows**	Body weight	kg	640	700	600	
	Mature weight	kg	640	700	600	
	DE^1^	%	69.8	67.4	69.2	
	NDF	%DM	nd	nd	nd	
	GEI^2^	MJ/head/d	424.40 ± 6.20^a^	357.20 ± 5.37^b^	368.30 ± 22.77^a,b^	<0.001
	Ym	%	5.7	6	5.8	
**Dry cows**	Body weight	kg	715	773	671	
	Mature weight	kg	715	773	671	
	DE	%	65	65	65	
	NDF	%DM	nd	nd	nd	
	GE^2^	MJ/head/d	175.80^b^	186.40^a^	167.60^a,b^	<0.001
	Ym	%	6.3	6.3	6.3	
**Heifers**	Body weight	kg	309	360	307	
	Mature weight	kg	640	700	600	
	ADG^3^	kg/d	1.09	1.30	1.09	
	DE^2^	%	65	65	65	
	NDF	%DM	nd	nd	nd	
	GE^2^	MJ/head/d	111^b^	122.80^a^	107.20^c^	<0.001
	Ym	%	6.3	6.3	6.3	

1 DE: Digestible Energy;

2 GE: Gross Energy Intake;

3 ADG: Average Daily Gain.

In the default-based approach, gross energy intake (GE) was estimated according to IPCC recommendations, using energy requirement equations adapted from the National Research Council (NRC 2001). Diet digestibility (DE) and neutral detergent fiber (NDF) values were drawn from default values provided by the Italian national emission inventory (ISPRA 2024). The CH_4_ conversion factor (Ym) was assigned according to the productivity level for lactating cows and feed characteristics for each animal category, as summarized in Table 1.

In the diet-based approach, GE was calculated using the actual gross energy content of the ration, and corresponding values for DE and NDF-derived from the ration composition analysis (Ratio-Best software; Table 2).

**Table 2:** Composition of representative diets for lactating cows, dry cows and heifers of the Holstein, Italian Red Pied and Brown Swiss breeds. Reported variables include dry matter intake (DMI), neutral detergent fiber (NDF), digestible energy (DE) and gross energy intake (GE). Values are presented as mean ± standard error. Statistically significant differences between breeds or physiological stages are indicated by superscript letters (*P* < 0.05).

Animal categories	Variables	Measure Units	Breeds			*P*-value
			Holstein	Italian Red Pied	Brown Swiss	
**Lactating cows**	DMI	kg/head/d	21.93 ± 0.26^a^	20.06 ± 0.52^b^	21.72 ± 0.46^ab^	0.001
	NDF	%	41.02 ± 0.68^a^	43.07 ± 1.12^a^	40.40 ± 3.47^a^	0.147
	DE^1^	%	68.25 ± 0.42^a^	65.44 ± 0.80^b^	70.34 ± 1.95^a^	0.001
	GE^2^	MJ/head/d	401.80 ± 4.66^a^	362.80 ± 9.40^b^	396.60 ± 8.07^a,b^	<0.001
	Ym	%	6.14	6.30	6.03	
**Dry cows**	DMI	kg/head/d	12.57 ± 0.43^a^	13.53 ± 0.81^a^	15.39 ± 1.21^a^	0.193
	NDF	%	53.22 ± 0.63^a^	53.99 ± 0.90^a^	53.80 ± 1.51^a^	0.838
	DE^1^	%	60.58 ± 0.41^a^	60.76 ± 0.43^a^	62.48 ± 1.25^a^	0.510
	GE^2^	MJ/head/d	223.49 ± 7.51^a^	241.90 ± 15.00^a^	276.70 ± 21.27^a^	0.168
	Ym	%	6.3	6.3	6.3	
**Heifers**	DMI	kg/head/d	10.12 ± 0.25^a^	10.90 ± 0.53^a^	9.83 ± 1.01^a^	0.658
	NDF	%	52.03 ± 0.58^a^	52.75 ± 0.81^a^	51.74 ± 1.98^a^	0.778
	DE^1^	%	61.57 ± 0.47^a^	61.25 ± 0.46^a^	66.72 ± 4.72^a^	0.659
	GE^2^	MJ/head/d	182.98 ± 4.34^a^	199.68 ± 9.15^a^	178.20 ±18.83^a^	0.557
	Ym	%	6.68	6.73	6.65	

1 DE: Digestible Energy;

2 GE: Gross Energy.

For both approaches, the CH_4_ outputs were expressed in three metrics: production (MeP; g/head/day) and yield (MeY; g CH_4_/kg DMI) for all animal categories and also intensity (MeI; g CH_4_/kg FPCM) for lactating cows. Additionally, MeI at the herd level was calculated by aggregating total herd CH_4_ emissions and dividing by the total FPCM produced at farm level.

### Statistical analysis

All data processing and statistical analyses were carried out in R v 4.2.2 (R Core Team 2024) using the packages FSA (Ogle et al. 2025), nortest (Gross and Ligges 2015), dplyr (Wickham et al. 2014; Kay and Wobbrock 2021), permuco (Frossard and Renaud 2021), and the base stats library. Normality was examined with the Shapiro–Wilk test; because most distributions deviated from data normality, exclusively non-parametric procedures were adopted. Overall breed differences were tested with the Kruskal–Wallis test and resolved, where significant, by Dunn’s post-hoc comparisons. Paired contrasts between emissions estimated under the default and diet-based approaches were performed with the Wilcoxon signed-rank test.

To disentangle the factorial effects of breed, diet, and their interaction on CH_4_-related traits while preserving the non-parametric structure of the data, an aligned-rank transformation (ART) factorial ANOVA was implemented with ARTool. The analytical model was:


$$Y_{\mathrm{ijkl}}=\mu +\alpha_{i}+\beta_{j}+{\left(\alpha \beta \right)}_{ij}+u_{l}+\varepsilon_{\mathrm{ijkl}}$$

where:

- *Y_ijkl_* is the aligned is the aligned-and-ranked animal-level observation for methane production (MeP), methane emission intensity (MEI), or methane yield (MeY), for the *l-th* animal fed the *k-th* diet in the *j-th* herd within the *i-th* breed
-
$\alpha_{i}$ is the fixed effect of the *i-th* breed (Holstein, Brown Swiss, Red Pied)
-
$\beta_{j}$ is the fixed effect of the *j-th* breed (Default Diet or data-based Diet)
-
${\left(\alpha \beta \right)}_{ij}$ is the fixed interaction effect between breed and diet
-
$u_{l}\;∼N(0,\;\sigma_{u}^{2})$ is the random effect of the *l-th* farm
-
$\varepsilon_{\mathrm{ijkl}}\;∼N(0,\;\sigma^{2})$ is the residual

The ART approach relaxes the assumptions of normality and homoscedasticity while maintaining the nominal Type I error rate under heteroscedastic, non-Gaussian conditions. Analytical rigor was reinforced by re-analyzing the identical model with an exact permutation ANOVA (aovperm, 9,999 random permutations), which produced empirical F-statistics and permutation-based *P*-value. Concordance between ART- and permutation-derived results was assessed; agreement across methods was taken as evidence of robust inference (Figures S1 and S2). Pairwise comparisons of farms within breeds and across animal categories (milking cows, heifers, dry cows) were performed for each CH_4_-related trait using estimates derived from both the Default-based and Diet-based approach. Farm-level differences were assessed using the Kruskal–Wallis test, followed by Dunn’s post-hoc test with Bonferroni and Benjamini–Hochberg (FDR) corrections for multiple comparisons.

## Results

### Farms description

Tables 2 and 3 present a summary of herd composition, milk productivity, and ration characteristics for the three dairy breeds under investigation.

**Table 3:** Herd composition and milk productivity data for the three main dairy breeds in Italy: Holstein, Italian Red Pied, and Brown Swiss. Data are expressed as mean ± standard error of the mean. Statistically significant differences between breeds in terms of average herd size and milk yield per farm are denoted by superscript letters (*P* < 0.05).

Animal category	Parameter	Measure units	Breeds			*P*-value
			Holstein	Italian Red Pied	Brown Swiss	
**Lactating cows**	Farms	n	96	34	6	
	Total head	n	6664	1128	380	
	Head	n/farm	69 ± 8^a^	33 ± 4^b^	64 ± 18^a,b^	0.002
	Milk yield	kg/head/d	32.22 ± 0.52^a^	24.71 ± 0.70^b^	27.48 ± 2.51^a,b^	<0.001
	FPCM^1^	kg /head/d	33.52 ± 0.78^a^	22.56 ± 0.87^b^	27.89 ± 3.32^a,b^	<0.001
**Dry cows**	Farms	n	82	25	5	
	Total head	n	904	206	62	
	Head	n/farm	10 ± 1	6 ± 1	11 ± 3	0.056
**Heifers**	Farms	n	95	34	6	
	Total sample head	n	5,943	956	264	
	Head	n/farm	62 ± 8^a^	28 ± 4^b^	44 ± 14^a,b^	0.007

1 FPCM: fat and Protein Corrected Milk.

Marked differences emerged among breeds in both average herd size and per-cow milk yield. Holstein farms maintained the largest herds, averaging 69 ± 8 lactating cows, followed by Brown Swiss with 64 ± 18, and Italian Red Pied with 33 ± 4 cows (*P* = 0.002). Holstein lactating cows recorded the highest daily milk yield (32.22 ± 0.52 kg milk/head/day) meanwhile the Italian Red Pied the lowest one (24.71 ± 0.70 kg milk/head/day; *P* < 0.001). Similarly, fat- and protein-corrected milk (FPCM) was significantly greater in Holstein cows compared to Brown Swiss animals (*P* < 0.001).

Breed-related differences were also evident in feed intake. Holstein lactating cows consumed significantly more dry matter (DMI; 21.93 ± 0.26 kg/head/day) and gross energy (GE) intake, reaching (401.80 ± 4.66 MJ/head/day) than Italian red Pied cows (*P* < 0.001). This finding was also confirmed in digestible energy (DE) value. In contrast, no significant breed-related differences were found in the nutritional parameters of dry cows and heifers, suggesting that feeding strategies for these categories were generally consistent across breeds.

Across all breeds, diets were primarily based on silage, though the extent of its use varied. Holstein cows had the highest average silage intake, consisting of 39.56 ± 20.82% of daily DMI, followed by Italian Red Pied (32.45 ± 24.62%) and Brown Swiss (30.98 ± 13.86%). Maize silage was the predominant forage component, contributing 31.32 ± 18.69% of DMI in Holsteins, 21.41 ± 18.27% in Brown Swiss, and 19.93 ± 20.69% in Italian Pied cows. Hay also played a substantial role in the ration, representing 26.81 ± 17.46% of DMI in Holsteins, 24.52 ± 22.27% in Brown Swiss, and 35.39 ± 22.11% in Italian Red Pied herds. Concentrates accounted for a variable share of the diet, with intake averaging 19.56 ± 13.46% of DMI in Holstein cows, 20.78 ± 14.35% in Italian Red Pied, and notably lower levels in Brown Swiss herds (11.00 ± 16.79%).

The diets of heifers and dry cows were predominantly hay-based, with only limited inclusion of silage and concentrates. Among heifers, hay represented the primary component of DMI, averaging 61.67 ± 25.20% in Holsteins, 64.50 ± 22.27% in Brown Swiss, and 70.72 ± 19.44% in Italian Red Pied. In contrast, silage, including maize silage, contributed only modestly to the ration, accounting for 20.08 ± 22.58% of DMI in Holsteins, 14.37 ± 17.15% in Brown Swiss, and 14.94 ± 19.54% in Italian Red Pied heifers. Concentrate inclusion was minimal across all breeds, with average values of 8.94 ± 13.59% DMI in Holsteins, 12.28 ± 15.58% in Brown Swiss, and 10.09 ± 12.30% in Italian Red Pied.

Similarly, the feeding strategy for dry cows was largely hay-based, with hay contributing 71.12 ± 23.10% of DMI in Holsteins, 74.38 ± 17.44% in Brown Swiss, and 76.89 ± 17.20% in Italian Red Pied herds. Silage inclusion remained low in this group as well, averaging 13.74 ± 19.53% of DMI in Holsteins, 6.73 ± 11.38% in Brown Swiss, and 11.30 ± 13.26% in Italian Red Pied. Maize silage contributed an even smaller fraction, representing just 10.17 ± 15.05% of DMI in Holsteins, 1.47 ± 3.30% in Brown Swiss, and 8.24 ± 10.87% in Italian Red Pied cows. Concentrate use in dry cow diets was uniformly low across breeds, averaging 8.58 ± 14.09% DMI in Holsteins, 4.25 ± 9.50% in Brown Swiss, and 6.69 ± 8.09% in Italian Red Pied.

### Enteric methane emissions

Table 4 summarizes breed-specific enteric CH_4_ emission estimates across quantification methods.

**Table 4:** Methane emission estimates for lactating cows, dry cows and heifers of Holstein, Italian Red Pied and Brown Swiss breeds based on two approaches: diet-based estimates and standard parameter assumptions. Reported variables include methane production (MeP, g/head/day), methane yield (MeY, g CH_4_/kg DMI) and methane intensity (MeI, g CH_4_/kg FPCM). Values are expressed as mean ± standard error. Statistically significant differences (*P* < 0.05) are indicated by superscript letters.

Animal categories	Variable	Measure Unit	Estimation approach	Breeds						*P*-value
				Holstein		Italian Red Pied		Brown Swiss		
					*P*-value		*P*-value		*P*-value	
**Lactating cows**	MeP^1^	g/head/d	Diet-based	443.60 ± 5.68^a^		410.92 ± 11.21^b^	*	429.45 ± 9.26^a,b^		0.009
			Default-based	437.02 ± 6.11^a^		385.25 ± 5.37^b^		382.10 ± 19.24^ab^		<0.001
	MeY^2^	g CH_4_/kg DMI	Diet-based	20.23 ± 0.10		20.48 ± 0.10	*	19.79 ± 0.34		0.051
			Default-based	20.18 ± 0.35		19.60 ± 0.49		17.58 ± 0.77		0.080
	MeI^3^	g CH_4_/kg FPCM	Diet-based	13.91 ± 0.39^a^	*	19.48 ± 1.08^b^		17.33 ± 3.31^a,b^		<0.001
			Default-based	13.40 ± 0.19^a^		18.03 ± 0.79^b^		14.77 ± 1.87^a^		<0.001
**Dry cows**	MeP^1^	g/head/d	Diet-based	253.01 ± 8.50	*	273.89 ± 16.98	*	313.21 ± 24.08		0.168
			Default-based	198.99^a^		210.99^b^		189.74^c^		<0.001
	MeY^2^	g CH_4_/kg DMI	Diet-based	17.75 ± 0.08		17.91 ± 0.05		18.00 ± 0.18		0.619
			Default-based	17.73 ± 0.83		16.97 ± 0.96		12.67 ± 1.10		0.147
**Heifers**	MeP^1^	g/head/d	Diet-based	220.17 ± 5.52	*	242.04 ± 11.39	*	213.13 ± 22.39		0.387
			Default-based	125.69± 0.00^a^		139.09± 0.00^b^		121.39± 0.00^c^		<0.001
	MeY^2^	g CH_4_/kg DMI	Diet-based	21.79 ± 0.17	*	22.27 ± 0.25	*	21.63 ± 0.47		0.197
			Default-based	13.33 ± 0.42		13.75 ± 0.65		13.21 ± 1.74		0.545
**Herd**	MeI^3^	g CH_4_/kg FPCM	Diet-based	21.25 ± 2.06^a^	*	29.99 ± 0.70^b^	*	26.02 ± 5.48^a,b^		0.0001
			Default-based	17.40 ± 0.34^a^		24.62 ± 1.44^b^		19.42 ± 3.33^a^		<0.001

1 MeP: Methane Production;

2 MeY: Methane Yield;

3 MeI: Methane Intensity. Asterisks indicate significant within-breed differences between diet-based and default-based estimates (Wilcoxon signed-rank test, *P* < 0.05).

**Figure 2:**
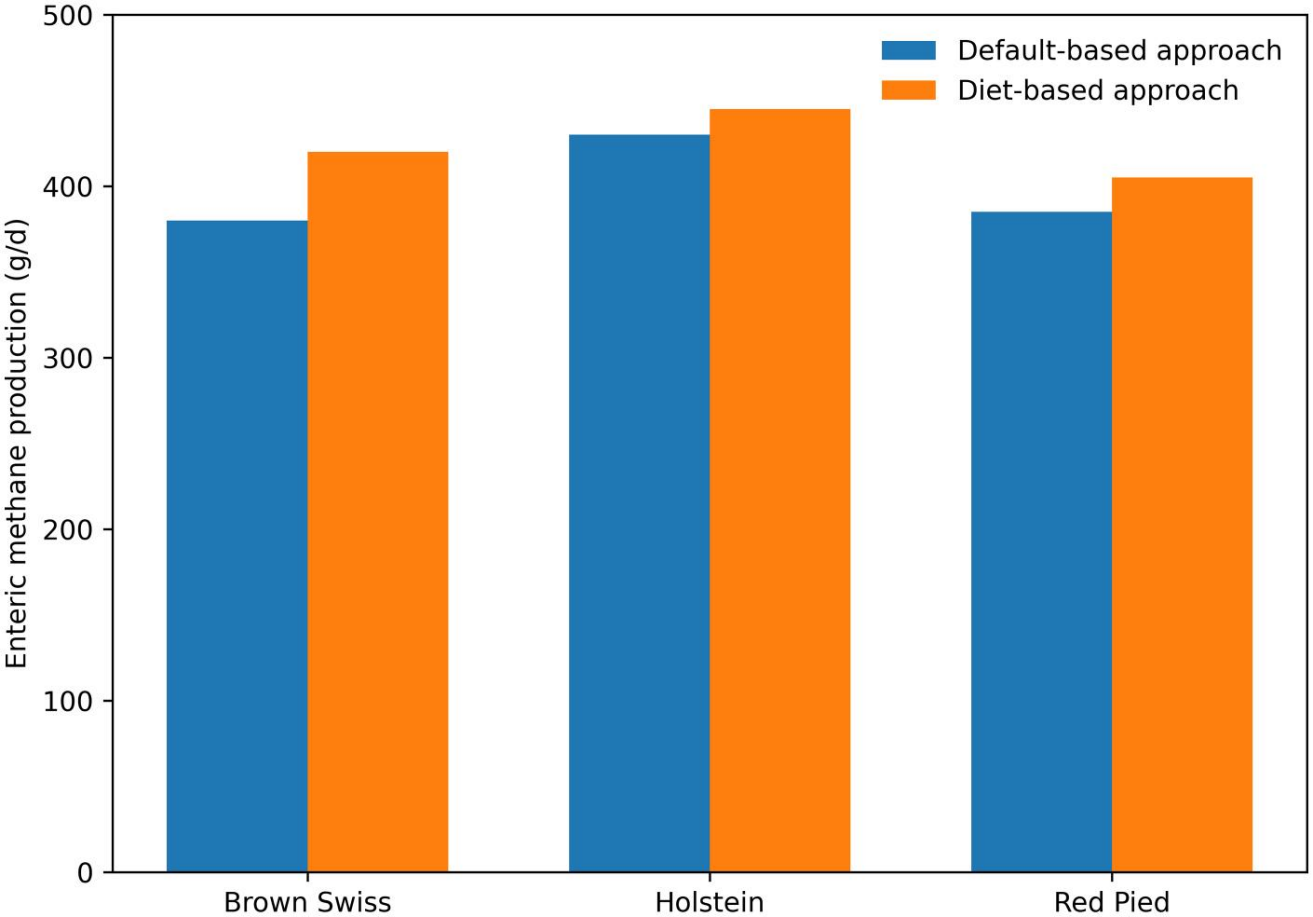
Bar plot showing average daily methane production (MeP_Lac, g/head/day) in lactating cows across three breeds (Brown Swiss, Holstein, and Italian Red Pied), estimated using either default dietary assumptions or farm-level dietary data.

Using the diet-based approach, Holstein lactating cows exhibited the highest methane production (MeP), averaging 443.60 ± 5.68 g CH_4_ g/head/day. This MeP was significantly greater than that of Italian Red Pied cows (410.92 ± 11.21 g; *P* < 0.05). Applying the default-based approach, the same breed hierarchy was maintained, but slightly lower absolute emissions were recorded for Italian Red Pied 385.25 ± 5.37 g (*P* < 0.05) (Figure 2).

Breed-related differences in methane yield (MeY) were not observed under the diet-based approach, with MeY values confined to a narrow range of 19.8 ± 0.3 to 20.5 ± 0.1 g/kg DMI. In contrast, the default-based approach revealed a significant lower MeY in Brown Swiss (17.58 ± 0.77 g/kg DMI) than in Holsteins (20.18 ± 0.35 g/kg DMI; *P* < 0.05).

In contrast, methane emission intensity (MeI) of lactating cows exhibited a different trend. With the diet-based approach, Italian Red Pied cows recorded the highest MeI (19.60 ± 0.49 g/kg FPCM), surpassing Holsteins (13.91 ± 0.39 g/kg FPCM; *P* < 0.05). The default-based approach confirmed this contrast (18.03 ± 0.79 vs. 13.40 ± 0.19 g/kg FPCM for Holstein and Red Pied respectively; *P* < 0.05).

Among dry cows, the diet-based approach revealed no breed effect: MeP ranged from 253.01 ± 8.50 to 313.21 ± 24.08 g/d with overlapping confidence intervals. When the default-based approach was applied, a significant difference emerged: Holsteins averaged 198.99 g/d, lower than Italian Red Pied (210.99 g/d) and Brown Swiss (189.74 g/d; *P* < 0.05). No significant effects of breed or estimation approach were detected for MeY.

For heifers, the diet-based approach revealed no statistically significant differences in MeP among breeds, with values ranging from 213.13 ± 22.39 for Brown Swiss to 242.04 ± 11.39 g/d for Italian Red Pied. Conversely, the default-based approach detected significant breed-specific variations, with Italian Red Pied (139.09 g/d) exhibiting greater CH_4_ emission compared to Holstein (125.69 g/d) and Brown Swiss (121.39 g/d; *P* < 0.05).

Under the diet-based approach, Italian Red Pied exhibited the greatest methane emission intensity (MeI) at the herd level (29.99 ± 0.70 g/kg FPCM), significantly higher than that of Holstein (21.25 ± 2.06 g/kg FPCM; *P* < 0.0001) and Brown Swiss herds (26.02 ± 5.48 g/kg FPCM). A similar pattern was observed under the default approach (*P* < 0.001), although MeI values were consistently lower than those estimated using the diet-based method (*P* < 0.05).

**Figure 3:**
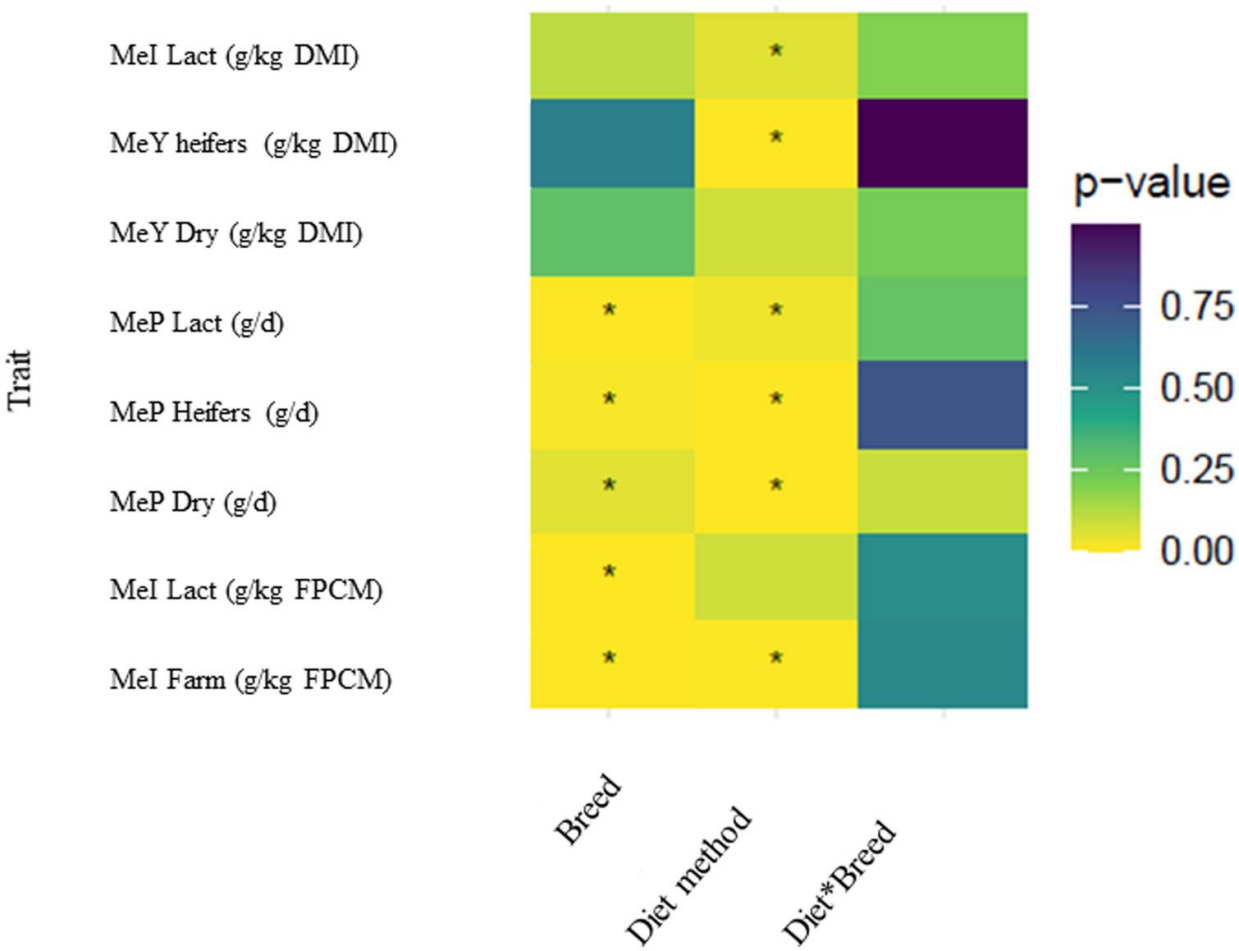
Heatmap of *P*value obtained from permutation tests following ART (Aligned Rank Transform) analysis, evaluating the effects of breed, diet method (default vs. real dietary data), and their interaction (diet × breed) on methane emission traits in dairy cows. Traits include methane yield (MeY), methane production (MeP), and methane emission intensity (MEI) across lactating cows (Lact), heifer, dry cows (Dry), and farm-level (Farm) contexts. Yellow cells indicate significant effects (*P* < 0.05), with asterisks (*) marking statistically significant comparisons.

Neither the global F-tests (Figure 3) nor the pair-wise post-hoc contrasts detected any biologically relevant Breed × Diet-based approach interaction. For every trait the permutation *P*value for the interaction exceeded 0.10, and none of the 63 pair-wise contrasts that decompose that term survived α  =  0.05 after multiplicity correction. The total of 63 contrasts arises as follows: a complete 3-breed × 2-method table contains six cells, giving 15 unique cell-to-cell comparisons; four trait–category combinations (MeP_Lactation, MeP_dry, MeY_Lactation and MeY_dry cows) were fully populated (4 × 15 = 60 contrasts), while MeP_heifer, missing one cell, provided the remaining three contrasts.

In empirical terms, breed ranking remained unchanged whether emissions were quantified with the default-based approach or with the diet-based one. Finally, the Dunn’s post-hoc pairwise comparisons of methane emission metrics (MeI, MeP and MeY) among farms within each cattle breed and animal categories, revealed a clear and consistent pattern of non-significance after adjustment for multiple comparisons.

## Discussion

Dry matter intake (DMI) estimated using the diet-based approach in this study (≈22 kg/day) aligned with values reported for mid-lactation Holstein cows managed under total mixed ration systems, which typically range between 20 and 25 kg/day (de Souza Congio et al. 2021). These estimates were also in agreement with projections from the NRC (2001) model, corresponding to approximately 3.5% of body weight (NRC 2001). The feeding strategies observed reflected breed-specific production systems. Holstein herds were primarily managed under intensive TMR-based regimens with moderate to high forage inclusion, whereas Brown Swiss herds relied predominantly on forage-based diets with limited concentrate supplementation (≈11% of DMI), consistent with extensive or semi-intensive systems. Similar feeding patterns have been reported for Brown Swiss cows in grazing-based systems, where concentrate intake typically accounts for 10%–15% of total DMI (Sabia et al. 2020). Italian Red Pied herds exhibited intermediate forage-to-concentrate ratios, reflecting their use in traditional or dual-purpose production systems and aligning with previous reports supporting moderate milk yields under such conditions (Renna et al. 2014). Consistent with these dietary patterns, Holstein cows exhibited greater DMI and gross energy intake (GEI) than Brown Swiss and Italian Red Pied cows. Both variables are well-established predictors of enteric methane emissions in lactating dairy cattle, with GEI explaining a large proportion of the variation in methane production across systems and DMI being closely associated with emission intensity when linked to productivity levels (Knapp et al. 2014; Appuhamy et al. 2016; Niu et al. 2018). Methane emission estimates obtained in this study were broadly consistent with values reported at European and global scales using proxy-based approaches and aligned with results from the METHAGENE project, which evaluated breed-specific emissions under standardized European conditions (Niu et al. 2018; Benaouda et al. 2019; Marumo et al. 2023; van Gastelen et al. 2023). This agreement supports the plausibility of the emission levels derived using both estimation approaches applied here. The breed-specific variation observed, with Holstein cows exhibiting the highest absolute methane production (MeP) but the lowest methane yield (MeY) and methane intensity (MeI), is consistent with previous findings in Italian dairy systems (Cassandro 2013; Martínez-Marín et al. 2023; Ferronato et al. 2025). This pattern likely reflects genetic differences arising from divergent selection histories. Intensive selection for milk yield and feed efficiency in Holsteins has increased intake capacity and productive output, resulting in higher total methane emissions but lower emissions per unit of milk produced (Berry et al. 2015). For lactating Holstein cows, both default- and diet-based estimates of MeP and MeY exceeded average daily enteric methane emissions reported at the European level (Appuhamy et al. 2016; Niu et al. 2018) and those previously reported for Italian dairy herds (Colombini et al. 2015, 2023). However, MeP estimates were comparable to values reported for Holstein cows in France whereas MeI values were lower, likely because of differences in milk productivity or dietary composition (Fresco et al. 2023). For Brown Swiss cows, diet-based estimates of methane production were greater than those reported in earlier Italian studies but fell within the broader range observed in other European studies (Bittante et al. 2018; Denninger et al. 2020). Despite the relatively high MeP, both MeY and MeI were lower than those reported by Denninger et al. (2020), suggesting differences in feed efficiency and production level. For Italian Red Pied cows, methane traits have been reported only sporadically, providing limited breed-specific benchmarks for external comparison. In this study, diet-based estimates of MeI exceeded values previously reported by Cassandro (2013), whereas default-based estimates were comparable, and MeY was consistent. The higher MeI under the diet-based approach likely reflects differences in productive performance or in the assumptions used to estimate DMI (Cassandro 2013). For dry cows and growing animals, diet-based estimates did not reveal significant breed differences in methane emissions, whereas the default-based approach suggested significant between-breed contrasts. This reduced variability may indicate limited precision in estimating DMI for nonproductive groups, likely affected by group feeding practices and herd-level management effects. At the same time, it may capture genuine on-farm conditions, where standardized feeding regimens are commonly applied across breeds and physiological stages (Schingoethe 2017). These findings underscore the importance of collecting high-resolution intake data to improve both the accuracy and discriminatory power of methane emission models for nonlactating animals. The absence of a significant Breed × Estimation Method interaction indicates that breed rankings were robust to the choice of estimation approach. Although default- and diet-based approaches produced different absolute emission values, relative differences among breeds remained consistent. This stability is particularly relevant for comparative assessments and supports the use of simplified approaches when detailed dietary data are unavailable, while also highlighting the added value of context-specific input data for accurate quantification. Identifying breeds with comparatively lower methane intensity is not intended to promote breed substitution as a mitigation strategy, but rather to support the development of breed-specific selection tools and management strategies aimed at improving feed efficiency and reducing emissions intensity within existing production systems.

## Conclusions

This study highlighted that breed-specific differences in enteric methane emissions persist across estimation approaches, while the magnitude of emissions is strongly influenced by the quality and resolution of dietary input data. Holstein, Brown Swiss, and Red Pied herds showed consistent breed rankings in methane production and intensity when estimated using both default- and diet-based approaches, indicating that simplified approaches are suitable for comparative assessments. In a dataset that had already been enhanced by DHI data, the incorporation of farm-level ration information still resulted in pronounced shifts in absolute methane estimates, most notably in non-productive groups, for which DHI-derived descriptors are least informative. This highlights the limited adequacy of relying on default assumptions alone. These findings underscore that accurate quantification of enteric methane emissions is driven less by the choice of estimation framework than by the precision of dietary inputs. Diet-based information enables a more realistic representation of on-farm conditions, reducing excessive over-standardization and enhancing the interpretability of methane inventories. At the same time, the robustness of breed rankings across methods suggests that default-based approaches can still offer informative guidance when detailed intake data are unavailable. Some limitations should be acknowledged. Dietary data were collected at a single time point and lacked detailed characterization of feed nutritional quality, which may have constrained the accuracy of intake and emission estimates. Strengthening data collection systems by incorporating longitudinal dietary records and improved feed characterization would improve the robustness of future analyses. In this context, the ongoing digital transformation of dairy systems offers opportunities to integrate automated data capture, standardized databases, and real-time monitoring, improving both data quality and scalability of methane assessments. Overall, coupling animal-based records with farm-specific dietary data provides a practical pathway for improving both the accuracy and representativeness of enteric methane estimates. By anchoring calculations to real management and feeding conditions, rather than generalized defaults, such approaches can support the development of more reliable inventories and inform mitigation strategies.

## Supplementary Material

skag171_Supplementary_Data

## Data Availability

The data underlying this article are available in the article and in its online supplementary material.

## Abbreviations

ADG: average daily gain
ART: aligned rank transform analysis
BW: body weight
CH4: methane
DE: digestibility
DHI: dairy herd improvements
DMI: dry matter intake
FPCM: fat and protein corrected milk
GE: gross energy
GEI: gross energy intake
IPCC: intergovernamental panel on climate change
MeI: methane intensity
MeP: methane production
MeY: methane yield
MIR: mid infrared
MW: mature weight
NDF: neutral detergent fiber
Ym: methane conversion factor
