# Supplementary material for: Breed-specific enteric methane emission factor assessment in Italian dairy cattle leveraging DHI and primary ration data
Source: J Anim Sci. 2026 Jun 2;104:skag171. doi: 10.1093/jas/skag171 (PMC13341014; doi:10.1093/jas/skag171)
Supplement: skag171_Supplementary_Data [file skag171_supplementary_data.docx]

**Supplementary materials**

**Supplementary Figure 1.** Log–log scatterplot comparing p-values derived from ART F-tests and those obtained via permutation testing across all traits and fixed effects. Each point represents a p-value pair for a specific trait-effect combination. The diagonal alignment of many points reflects general agreement in significance levels; however, deviations—particularly in the lower p-value range—highlight discrepancies in stringency between methods. Notably, ART F-tests tend to yield smaller (more liberal) p-values, identifying more effects as statistically significant, while permutation tests are generally more conservative. The divergence emphasizes the importance of permutation-based inference for controlling false positives in non-parametric factorial analyses.

**
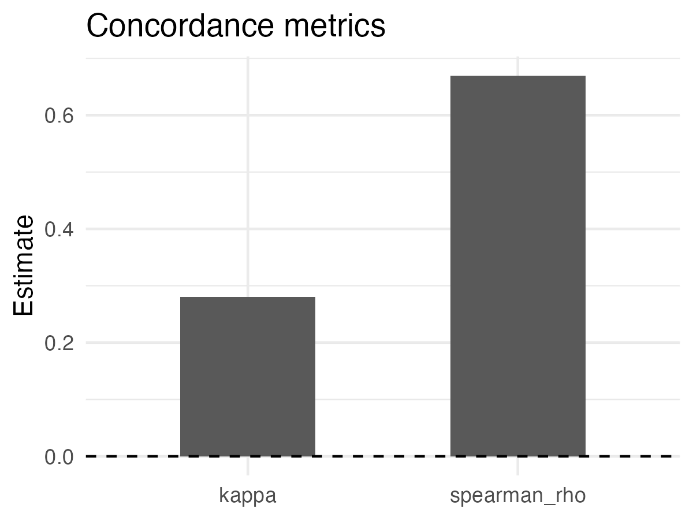
**

**Supplementary Figure 2.** Bar plot showing two concordance metrics between p-values obtained from ART-based F-tests and permutation tests. Cohen’s kappa (κ = 0.29) reflects agreement in binary significance classification (p < 0.05), indicating only moderate overlap in identifying significant effects. Spearman’s rank correlation (ρ = 0.68) reveals stronger agreement in the relative ranking of p-values across traits and effects. The relatively low κ suggests that permutation tests tend to be more conservative, flagging fewer effects as significant compared to ART F-tests. This difference highlights the importance of using permutation-based p-values for robust inference in non-parametric factorial designs, especially when controlling for Type I error.
